# Prediction of miRNA-disease associations in microbes based on graph convolutional networks and autoencoders

**DOI:** 10.3389/fmicb.2023.1170559

**Published:** 2023-04-28

**Authors:** Qingquan Liao, Yuxiang Ye, Zihang Li, Hao Chen, Linlin Zhuo

**Affiliations:** ^1^College of Computer Science and Electronic Engineering, Hunan University, Changsha, China; ^2^School of Data Science and Artificial Intelligence, Wenzhou University of Technology, Wenzhou, China; ^3^School of Computing and Data Science, Xiamen University Malaysia, Sepang, Selangor, Malaysia

**Keywords:** miRNA-disease association, microbial ecology, dual-autoencoder, graph convolutional network, insufficient information, topological information, robust representations

## Abstract

MicroRNAs (miRNAs) are short RNA molecular fragments that regulate gene expression by targeting and inhibiting the expression of specific RNAs. Due to the fact that microRNAs affect many diseases in microbial ecology, it is necessary to predict microRNAs' association with diseases at the microbial level. To this end, we propose a novel model, termed as GCNA-MDA, where dual-autoencoder and graph convolutional network (GCN) are integrated to predict miRNA-disease association. The proposed method leverages autoencoders to extract robust representations of miRNAs and diseases and meantime exploits GCN to capture the topological information of miRNA-disease networks. To alleviate the impact of insufficient information for the original data, the association similarity and feature similarity data are combined to calculate a more complete initial basic vector of nodes. The experimental results on the benchmark datasets demonstrate that compared with the existing representative methods, the proposed method has achieved the superior performance and its precision reaches up to 0.8982. These results demonstrate that the proposed method can serve as a tool for exploring miRNA-disease associations in microbial environments.

## 1. Introduction

MiRNAs are a class of endogenous short RNAs that have multiple important regulatory functions in the microbial environment. MiRNAs exert a significant influence in microbial ecology such as metabolism (Karp and Ambros, 2005), cell growth (Ambros, 2003), immune response (Jung et al., 2006), proliferation (Miska, 2005), cell cycle regulation (Liu et al., 2022a) and tumor invasion (Meng et al., 2007). Moreover, miRNAs completes the process of regulating gene expression by base-pairing with target RNA (Jopling et al., 2005; Vasudevan et al., 2007). As a result, miRNAs can effectively predict the occurrence of diseases in microbial ecology and contribute in prevention and diagnosis. HMDD and Human Cancer Differentially Expressed miRNA Database (dbDEMC) contains miRNA-disease related information (Li et al., 2014). However, the data available for research are relatively scarce, and the choice of wet assays to determine miRNA-disease associations is expensive. Thus, it is crucial to design an effective model to handle the experimental testing process (Chen et al., 2019a, 2021; Wang et al., 2019; Zhu et al., 2021).

In the field of biocomputing, correlation studies between various molecules have been conducted. For example, researchers predict the interaction between circRNA and disease (Wang et al., 2021), miRNA and lncRNA (Zhang et al., 2021), lncRNA and protein (Hu et al., 2018), etc. The aforementioned methods are necessary to predict miRNA-diseases, and most of them are based on complex networks. This line of research works builds one or multi networks on the original interaction datasets, and predicts disease-related miRNAs by integrating multi-level data. In general, these approaches can make reasonable predications about miRNA relatedness based on similar disease phenotypes and similar functions, and vice versa (You et al., 2017; Chen et al., 2018a,d, 2019b). For instance, Jiang et al. established a scoring mechanism for predicting disease-miRNA correlations based on miRNA-disease heterogeneous networks, and applied hypergeometric distribution to predict the strength of miRNA-disease associations (Jiang et al., 2010). Guided by global information of the data, Chen et al. proposed a strategy based on random walk to predict the association between diseases and miRNAs (Chen et al., 2012). Considering the fact that most of models cannot accurately predict miRNAs associated with isolated disease individuals, Zeng et al. added some perturbations to the network to train the predictor (Zeng et al., 2018). Recently, researchers have explored a wide range of miRNA functions, which increases the complexity of analyzing gene expression and regulatory networks in common diseases today (Vickers et al., 2014). Moreover, studies have shown that miRNAs participate in the regulation of many cardiovascular-related diseases. These studies demonstrate new aspects of miRNAs in the field of life sciences, and analyzing the regulation of these miRNAs on cardiovascular-related diseases is extremely valuable for proposing new diagnostic and preventive strategies.

Some studies based on statistical methods to predict miRNA-disease associations are attracting more and more attention from the researchers. For example, Li et al. constructed an SVM classifier based on miRNAs associated with specific tumor phenotypes (Li et al., 2012). This model is only for the prediction of diseases such as tumors and may not be suitable for other diseases. Considering the shortage of negative samples in supervised learning models, Yan et al. proposed a model that can reveal the interaction between diseases and miRNAs based on the principle of regularized least squares (Chen and Yan, 2014). This model can predict the associated miRNAs of emerging diseases, thanking to its semi-supervised learning strategy. Chen et al. demonstrated a computational model of matrix decomposition and heterogeneity network inference for predicting miRNA-disease associations (Chen et al., 2018c). In this model, similarities in disease signatures and disease-miRNA associations are integrated into a unified network. However, model parameters are relatively large, and how to reasonably set the parameters is a very challenging task. Xu et al. developed a novel model based on probabilistic matrix factorization (Xu et al., 2019). This model firstly integrates the similarity in the miRNA-disease network; And then performs a probability matrix factorization operation based on the interaction matrix and the similarity matrix.

However, the aforementioned models cannot still achieve promising performance in predicting miRNA-disease associations. Note that deep learning technology has recently been applied to the field of biological computing (Fu et al., 2020; Cai et al., 2021a,b; Liu et al., 2022c; Peng et al., 2022a,b,c; Tian et al., 2022; Xu et al., 2023; Zhang et al., 2023). For instance, Chen et al. constructed a restricted Boltzmann model that can predict associations in different domains (Chen et al., 2015). Because the variability among multiple types cannot be fully modeled, the prediction accuracy is not promising. Chen et al. pre-trained all miRNA-disease pairs on a restricted Boltzmann model and fine-tuned on DBN on the same proportion of positive and negative samples to obtain prediction scores (Chen, 2021). Peng et al. extract features based on a three-autoencoder and then apply a convolutional network to predict the final label (Peng et al., 2019).

Recently, graph neural networks have received much attention from the researchers. For instance, Chen et al. developed a method for miRNA disease association determination based on heterogeneous graphs (Vickers et al., 2014). Furthermore, Chen et al. proposed a network-integrated miRNA-disease-associated internal and external score prediction method (Chen and Zhang, 2014). Chen et al. proposed a predictive model integrating matrix deconstruction and heterogeneous graph aggregation (Chen et al., 2016). Chen et al. utilized matrix factorization to alleviate the influence of noise in adjacent matrices, and then perform node aggregation operations on heterogeneous networks. Mugunga proposed a predictive model based on path features and random walk to obtain correlation scores for miRNA-associated diseases, and potential miRNA-disease associations would be associated with high prediction scores (Mugunga et al., 2017). Guo et al. used a decision fusion strategy to prioritize the results of existing methods, and then verified the effectiveness of the decision fusion strategy (Guang, 2018). Zeng et al. constructed a heterogeneous network to predict potential associations between miRNAs and disease, while also accounting for dataset imbalance (Zeng, 2017). The model also uses a multi-layer perceptron-based approach to predict miRNA-disease pairs, integrating a variety of biological data resources.

Although the aforementioned methods are outstanding in predicting miRNA-disease associations, few studies consider the similarity and topological information comprehensively. Generally speaking, when the topological structure is very sparse, feature information becomes more important in association prediction; when feature information is incomplete, topological information can also play an auxiliary role. Inspired by this guidance, we propose a GCN and autoencoder-based approach that can comprehensively consider both feature and topological information in miRNA-disease networks. Our contributions can be summarized as follows:

1. We develop a GCNA-MDA model to predict miRNA-disease association based on GCN and autoencoders, which achieves the excellent performance. We employ dual-autoencoders to extract disease and miRNA features, which improves the robustness of node presentation. At the same time, we apply a 2-layer GCN to further aggregate disease and miRNA node features by fully considering the topological information.
2. We propose a robust strategy for constructing miRNA and disease basic feature matrix. Combining feature similarity and Gaussian similarity, a unified similarity matrix is constructed. Adding association information to the disease and miRNA nodes respectively make the feature representation more abundant, thus alleviate the negative impact of insufficient data.
3. We conduct multiple comparison experiments on the HMDD dataset to verify that the GCNA-MDA model can accurately perform the prediction task. Moreover, we construct case studies to verify that the GCNA-MDA model can indeed be applied to examine the specific miRNA-disease associations.

## 2. Materials and methods

### 2.1. Dataset

The dataset used in the experiment could be downloaded from the HMDD v2.0 database (Li et al., 2014). The dataset includes 5430 validated associations generated by 495 miRNAs and 383 diseases. It can be abbreviated as adjacency matrix *A*, in which there are 495 × 383 miRNA disease associations. If disease *d* is associated with miRNA *m*, the association relationship is satisfied, that is, *A*(*m, d*) = 1, otherwise its value is 0.

### 2.2. Constructing miRNA and disease basic feature matrix

In this section, we describe in detail the process of constructing robust initial feature for miRNAs and diseases. These similarity matrices can be used as the input matrices for the autoencoder in the next stage. The main process will be introduced below.

#### 2.2.1. Disease feature similarly

Based on the collected disease original feature information, its feature similarity network can be constructed (Schriml et al., 2012). Specifically, we apply the strategy of DAG to denote these diseases. For a disease node *d*, it is denoted by *DAG*(*d*) = (*d, v*(*d*), *e*(*d*)). *v*(*d*) represents the set of nodes reached to *d*, and *e*(*d*) represents all edges linked to *d*. In the DAG graph, the feature contribution weight *W* of the upper node *x* to *d* is calculated as follows:

(1) $$\begin{array}{l} W1_{d}\left(x\right)=\{\begin{array}{ll} 1\; & if\;x=d\\ max\left\{▽*W1_{d}\left(x^{\prime }\right)|x^{\prime }\in x_{children}\right\}\; & if\;x\neq d, \end{array} \end{array}$$

where ▽ represents the adjustment parameter of *W*, which is empirically set to 0.5 (Chen and Yan, 2013). Based on *d* and its upper nodes, the feature representation value of *d* can be calculated as follows:

(2) $$\begin{array}{l} Df1\left(d\right)=\underset{x\in v\left(d\right)}{\sum }W1_{d}\left(x\right). \end{array}$$

We hypothesize that the greater the number of DAGs shared between two disease nodes, the smaller the difference between the two nodes may be. Thus, the feature similarity of two disease nodes *A* and *B* can be calculated as:

(3) $$\begin{array}{l} FS1\left(A,B\right)=\frac{\sum_{x\in v\left(A\right)⋂v\left(B\right)}W1_{A}\left(x\right)+W1_{B}\left(x\right)}{Df1\left(A\right)+Df1\left(B\right)} \end{array}$$

For disease node *d*, if two nodes involve approximately the same *DAG*(*d*) level, then two nodes should have different occurrence ratios and their contribution to the feature weight of disease *d* should be different. Thus, we propose the following equation to compute the influence of disease *x* on *d*:

(4) $$\begin{array}{l} W2_{d}\left(x\right)=-log\frac{\left|DAG\left(x\right)\right|}{\left|D\right|}, \end{array}$$

where *D* denotes the disease set, and |·| denotes the operation of calculating the number of elements in the set. Similarly, the feature representation value of *d* and the feature similarity of two disease nodes *A* and *B* can be calculated as Equations 5 and 6, respectively:

(5) $$\begin{array}{l} Df2\left(d\right)=\underset{x\in v\left(d\right)}{\sum }W2_{d}\left(x\right), \end{array}$$

(6) $$\begin{array}{l} FS2\left(A,B\right)=\frac{\sum_{x\in v\left(A\right)⋂v\left(B\right)}W2_{A}\left(x\right)+W2_{B}\left(x\right)}{Df2\left(A\right)+Df2\left(B\right)}. \end{array}$$

Combining the two measure methods to obtain a more reasonable feature similarity, the calculation equation is as follows:

(7) $$\begin{array}{l} FS\left(A,B\right)=\frac{FS1\left(A,B\right)+FS2\left(A,B\right)}{2}. \end{array}$$

#### 2.2.2. Similarity based on Gaussian

We hypothesize that two miRNAs with small functional differences should be associated with diseases with similar properties (Van Laarhoven et al., 2011). Based on this assumption, we apply the Gaussian kernel distance calculation equation to calculate the similarity between disease nodes *D*_*a*_ and *D*_*b*_:

(8) $$\begin{array}{l} GD\left(D_{a},D_{b}\right)=exp\left(-\gamma_{d}∥Index\left(D_{a}\right)-Index\left(D_{b}\right){∥}^{2}\right), \end{array}$$

where

(9) $$\begin{array}{l} -\gamma_{d}=-\gamma_{d}^{\prime }\left(\frac{1}{\left|D\right|}\overset{\left|D\right|}{\underset{i=1}{\sum }}{∥Index\left(D_{i}\right)∥}^{2}\right), \end{array}$$

and γ_*d*_ represents the Gaussian kernel parameter, and represents the index function, which can index the row vector of the matrix. Similarly, the Gaussian kernel distance formula between miRNA nodes *miR*_*a*_ and *miR*_*b*_ is as follows:

(10) $$\begin{array}{l} GM\left(miR_{a},miR_{b}\right)=exp\left(-\gamma_{m}∥Index\left(miR_{a}\right)-Index\left(miR_{b}\right){∥}^{2}\right), \end{array}$$

where

(11) $$\begin{array}{l} -\gamma_{m}=-\gamma_{m}^{\prime }\left(\frac{1}{\left|M\right|}\overset{\left|M\right|}{\underset{i=1}{\sum }}{∥Index\left(miR_{i}\right)∥}^{2}\right), \end{array}$$

and *M* represents the miRNA node set, and γ_*d*_ and γ_*m*_ are often set to 1 empirically (Chen and Yan, 2013).

#### 2.2.3. Similarity integration

Due to missing data, some disease pairs may not exist in the feature similarity. For this case, using Gaussian kernel distance to measure the distance between diseases can robustly reflect the differences between diseases. Therefore, the calculation formula of the overall similarity between disease nodes *A* and *B* is formulated as

(12) $$\begin{array}{l} SD\left(A,B\right)=\{\begin{array}{ll} \frac{GD\left(A,B\right)\;+\;FS\left(A,B\right)}{2}\; & if\;x=d\\ GD\left(A,B\right)\; & if\;x\neq d. \end{array} \end{array}$$

Similarly, the calculation equation of the overall similarity between miRNA nodes *X* and *Y* is representated as follows:

(13) $$\begin{array}{l} SM\left(X,Y\right)=\{\begin{array}{ll} \frac{GM\left(X,Y\right)\;+\;FM\left(X,Y\right)}{2}\; & if\;FM\left(X,Y\right)\;exists\\ GM\left(X,Y\right)\; & otherwise, \end{array} \end{array}$$

where *FM*(·, ·) denotes the functional similarity score between two miRNA nodes.

### 2.3. Model design

**Figure 1:**
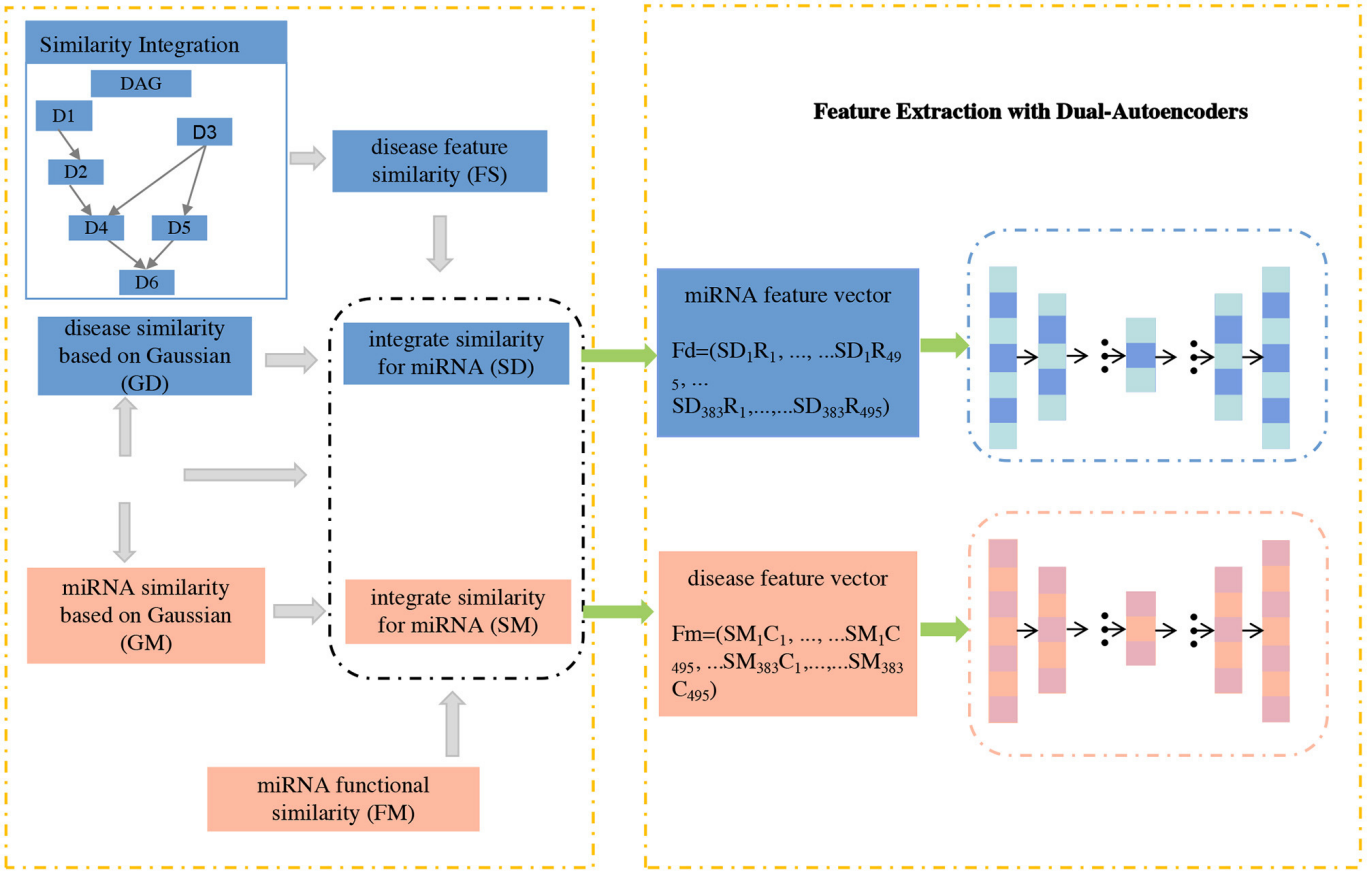
The concatenated feature representations are input to dual-autoencoders.

In this section, we propose GCNA-MDA model for predicting miRNA-disease associations based on GCNs and dual-autoencoders. It mainly consists of three parts: firstly, a new similarity calculation strategy is used to obtain the initial basic feature matrix of miRNA (or disease); secondly, a dual-autoencoder is applied to extract the robust expression of miRNA and disease respectively; finally, a 2-layer GCN is applied to predict miRNA-disease associations. Next, the GCNA-MDA model architecture will be introduced in detail, and its overall framework is shown in Figure 1.

#### 2.3.1. Node representation

In this subsection, a novel signature expression for miRNA (or disease) nodes is proposed. Considering that the direct interaction information between miRNA and disease is very important, we add disease-related information to the features of miRNA nodes. Similarly, we also add the corresponding miRNA information to the disease node. Specifically, according to formulas (13) and (12), we calculate the respective feature vectors based on miRNAs and diseases, respectively. Based on the above formula, the fusion with the miRNA-disease association matrix can be obtained:

(14) $$\begin{array}{l} F_{d}={\left(SD_{1}R_{1},...,SD_{1}R_{495},...,SD_{383}R_{1},...,SD_{383}R_{495}\right)}^{T}, \end{array}$$

(15) $$\begin{array}{l} F_{m}={\left(SM_{1}C_{1},...,SM_{1}C_{495},...,SM_{383}C_{1},...,SM_{383}C_{495}\right)}^{T}, \end{array}$$

where *R*_*i*_ and *C*_*j*_ represent the *i*−*th* row and *j*−*th* column vectors of the miRNA-disease association matrix, respectively. Subsequently, the matrices *F*_*m*_ and *F*_*d*_ of miRNAs and diseases were fed into a dual-autoencoder, respectively.

#### 2.3.2. Feature extraction with dual-autoencoders

Based on the above presentation, the node expression of the miRNA (or disease) node fused with the correlation relationship can be obtained. Obviously, the number of nodes is small (383 and 495), but the vector length of each node is high (equal to twice the number of nodes of each type). In this case, the deep neural network may suffer from insufficient samples. Fortunately, autoencoders can play their unique role in this situation. With the strategy of unsupervised learning, the automatic encoding machine no longer needs a large number of samples for its training. This is convenient for us to extract more robust features for the next stage of association prediction tasks.

We extract features of miRNAs and disease nodes separately based on a symmetric dual-autoencoder. The process is mainly divided into two stages of encoding and decoding. During the encoding phase, the basis vectors of the nodes obtained in the previous section is fed into the encoder network. By setting a reasonable number of dimensions, low-rank feature vectors of miRNAs and diseases can be obtained. The calculation method in the encoder is:

(16) $$\begin{array}{l} Y=\sigma_{e}\left(W_{e}X+b_{e}\right), \end{array}$$

where σ_*e*_() represents the sigmod activation function. *W*_*e*_ and *b*_*e*_ represent the weight and bias matrices in the encoder, respectively. Both matrices can be efficiently trained in the encoder. Thus, the low-rank vectors obtained from the encoding stage are fed into the decoder network. By setting a reasonable number of dimensions, robust feature vectors for miRNAs and diseases can be obtained. The calculation method in the decoder is:

(17) $$\begin{array}{l} F=\sigma_{d}\left(W_{d}X+b_{d}\right), \end{array}$$

where σ_*d*_(·) represents the sigmod activation function. *W*_*d*_ and *b*_*d*_ represent the weight and bias matrices in the decoder, respectively. *F* is stored as the final feature vector and is fed to the GCN in the next stage for association prediction tasks. To minimize the final feature distribution and the node's initial basic feature distribution, an optimization objective of the dual-autoencoder can be set as:

(18) $$\begin{array}{l} Loss=\underset{x\in X}{\sum }∥x-F_{x}{∥}^{2}. \end{array}$$

In our research, we apply the common square loss function as the optimization objective. The *X* matrix covers all miRNA and disease nodes, and *x* is a row vector in the *X* matrix, which can be regarded as a certain node. In the last layer of the decoder, the node vector length is empirically set to 128.

#### 2.3.3. Predict miRNA–Disease association by GCN

Through the aforementioned process, we can obtain robust features of miRNAs and disease nodes. It is well known that graph neural networks can well aggregate node features and fully consider the topological information of miRNA-disease networks. Therefore, this study uses GCN to predict whether there is an association between miRNA nodes and disease nodes. Since GCN is suitable for tasks on graphs with only one type of nodes and one type of links. Therefore, in order to obtain a unified node adjacency matrix, it is necessary to splice miRNA nodes and disease nodes. For adjacency matrix *A*, the first 495 indexes of its row (or column) represent miRNA, and the last 383 indexes represent disease. For the elements in the matrix, the sub-matrix composed of elements from 1 to 495 rows and 496 to 878 columns represents miRNA-disease association. The specific calculation is as follows:

(19) $$\begin{array}{l} A=\left(\begin{array}{cc} N_{MM} & N_{MD}\\ N_{DM} & N_{DD} \end{array}\right). \end{array}$$

**Figure 2:**
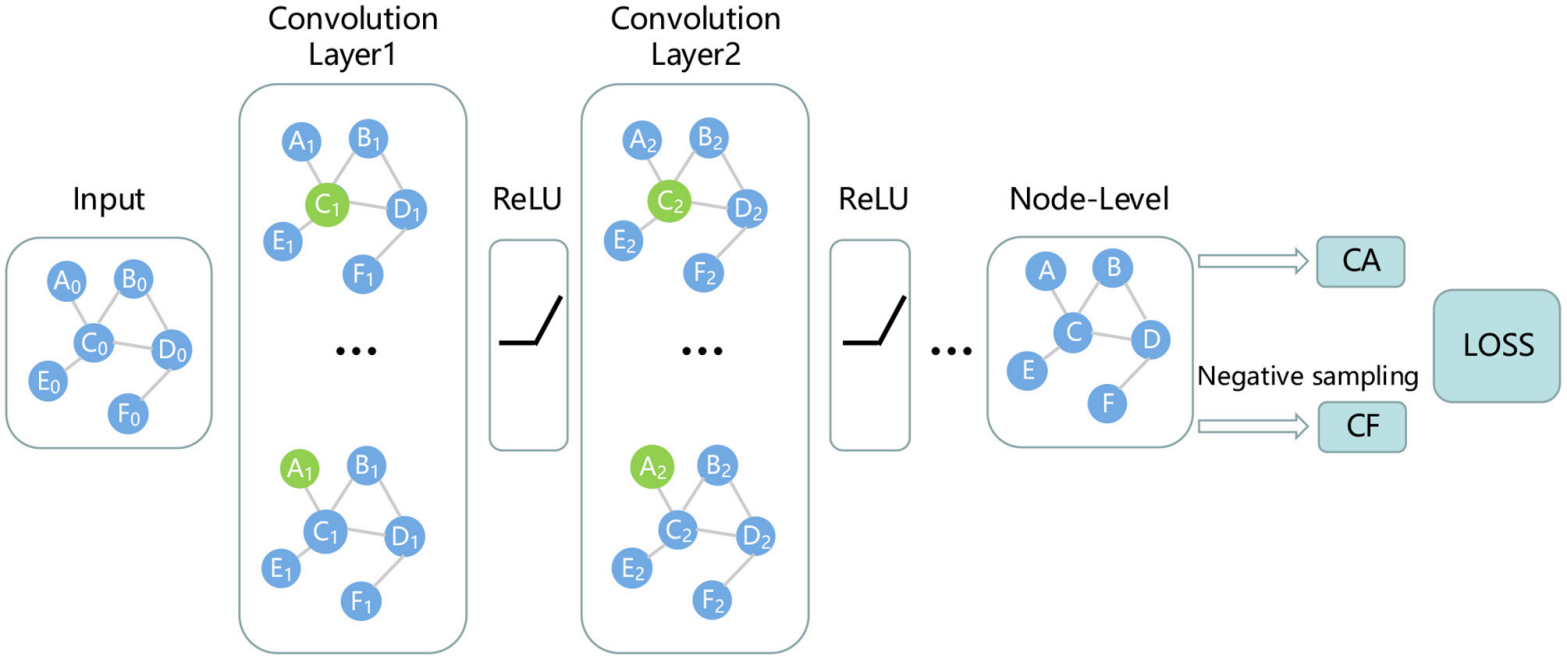
Prediction of miRNA-disease associations using GCN.

In the above equation, the size of the adjacency matrix *A* is 878 × 878. *N*_*MD*_ and *N*_*DM*_ represent miRNA-disease association, and *N*_*DD*_ and *N*_*MM*_ are set to 0. In GCN, the feature matrix *F* obtained in the previous section is fed into the GCN network as the initial node embedding matrix. Along with it, matrix *A* participates in GCN. GCN can aggregate nodes based on topology information to obtain more effective node embedding. The node embedding aggregation calculation is as follows:

(20) $$\begin{array}{l} H_{i+1}=\sigma \left({\hat{\Gamma }}^{-\frac{1}{2}}Â{\hat{\Gamma }}^{-\frac{1}{2}}H_{i}W_{i}\right), \end{array}$$

where *H*_*i*_ represents the node embedding of the *i*-th layer, *H*_0_ comes from *Fd* or *Fm*. Â represents the adjacency matrix with self-loops, and $\hat{\Gamma }$ represents the degree matrix of Â, *W*_*i*_ represents the trainable matrix. In this study, we design a 2-layer GCN to predict miRNA-disease associations as shown in Figure 2.

## 3. Results

In this section, our model compares the performance of several typical models on the HMDD dataset. In order to verify the reliability of the model, we also conducted 5-fold and 10-fold cross-validation experiments. At the same time, to demonstrate that the proposed model has certain practical significance, such as preliminary prevention and guidance for diseases, we also constructed corresponding case studies for certain diseases.

### 3.1. Evaluation strategy

We used common AUC and precision metrics to validate the performance of our model. Among them, AUC is a comprehensive indicator, which can reflect the comprehensive performance of the model. Since the sparse rate in the dataset is ((495*X*383) − 5430) ÷ (495*X*383) ≈ 97.14%, in other words, the number of negative samples is far more than that of positive samples. However, from a practical point of view, we need to pay more attention to the performance of the model in the positive sample. Therefore, we use Precision to evaluate the performance of the model. Its calculation formula is as follows:

(21) $$\begin{array}{l} Precision=\frac{True\;Positive\;rate}{True\;Positive\;rate\;+\;False\;Negative\;rate}. \end{array}$$

Furthermore, in *N*-fold cross-validation experiments, we perform *N*-fold cross-validation by randomly splitting the sample into *N* equal parts. *N* − 1 parts are used as the training set, and the rest are used as the test set. According to this strategy, *N* parts are used in turn as test sets, and the remaining parts are used as training sets to complete all cross-validation experiments. In the experiment, we consider the AUC metric to measure the performance of the model.

### 3.2. Comparative evaluation

We compare the GCNA-MDA model with GAEMDA (Li et al., 2021), GBDT_LR (Zhou et al., 2020), ABMDA (Zhao et al., 2019), LMTRDA (Wang et al., 2019), RFMDA (Chen et al., 2018b) models. The GAEMDA (Li et al., 2021) model fuses similarity information and topological neighborhood information in the miRNA-disease network, and integrates GCN and autoencoder for prediction tasks. GBDT_LR (Zhou et al., 2020), ABMDA (Zhao et al., 2019) and RFMDA (Chen et al., 2018b) use ensemble learning strategies to obtain high-quality features and then make corresponding predictions. Besides, GBDT_LR (Zhou et al., 2020), ABMDA (Zhao et al., 2019) used a new negative sample collection strategy to weaken the impact of negative sample coverage. LMTRDA (Wang et al., 2019) combined multi-way data for prediction tasks. Table 1 lists the results of performance comparison, indicating that the GCNA-MDA model obtains the highest Precision value of 89.82%. Our model fully incorporates multi-level information, while applying a dual-autoencoder to further refine the features. Meanwhile, we applies GCN to predict miRNA-disease associations, making the good use of topological information. Combining the above two reasons, our model has achieved the best accuracy results.

**Table 1:** Precision of six methods in miRNA-disease classification task.

**Models**	**Precision (%)**
RFMDA (Chen et al., 2018b)	62.53
LMTRDA (Wang et al., 2019)	80.13
ABMDA (Zhao et al., 2019)	81.52
GAEMDA (Li et al., 2021)	81.37
GBDT_LR (Zhou et al., 2020)	83.15
GCNA-MDA	87.80

For the compared models, RFMDA (Chen et al., 2018b) achieves the worst performance. The main reason is attributed that although the model adopts the strategy of integrated learning, RFMDA (Chen et al., 2018b) does not consider the skew caused by excessive negative samples and it does not synthesize information from multiple sources. While the rest of the models employing multiple information significantly outperform the RFMDA (Chen et al., 2018b) model, which exhibits the importance of integrating multiple information. In addition, GBDT_LR (Zhou et al., 2020) combined with ABMDA (Zhao et al., 2019) applied the strategy of ensemble learning and weakening negative samples, resulting in a significant performance improvement.

### 3.3. Scalability evaluation

**Figure 3:**
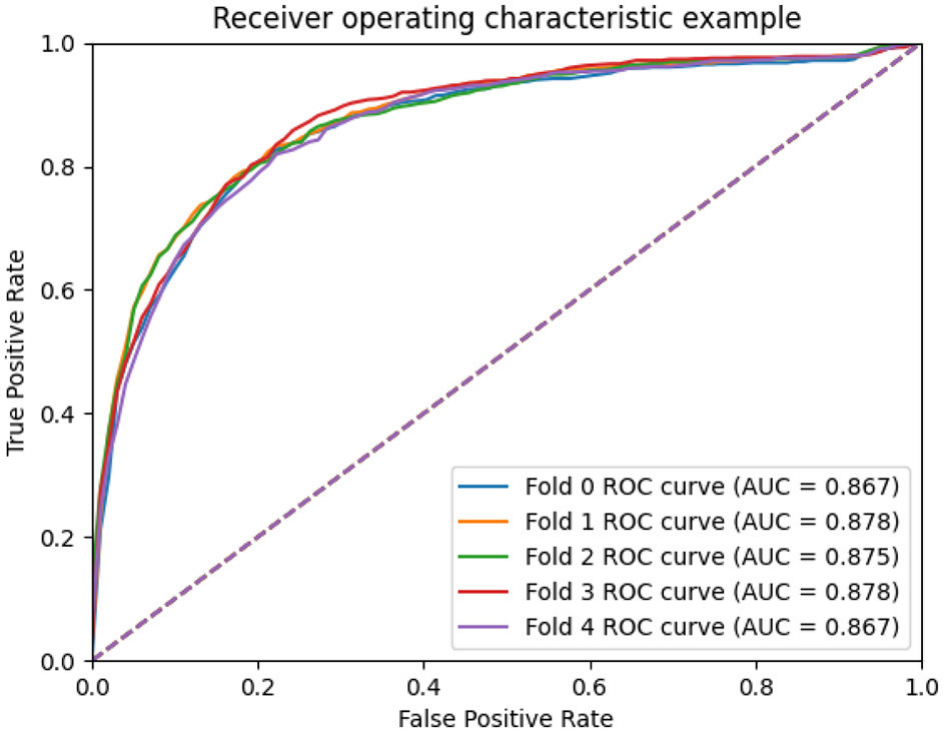
AUC performance of GCNA-MDA model on 5-fold cross-validation.

**Figure 4:**
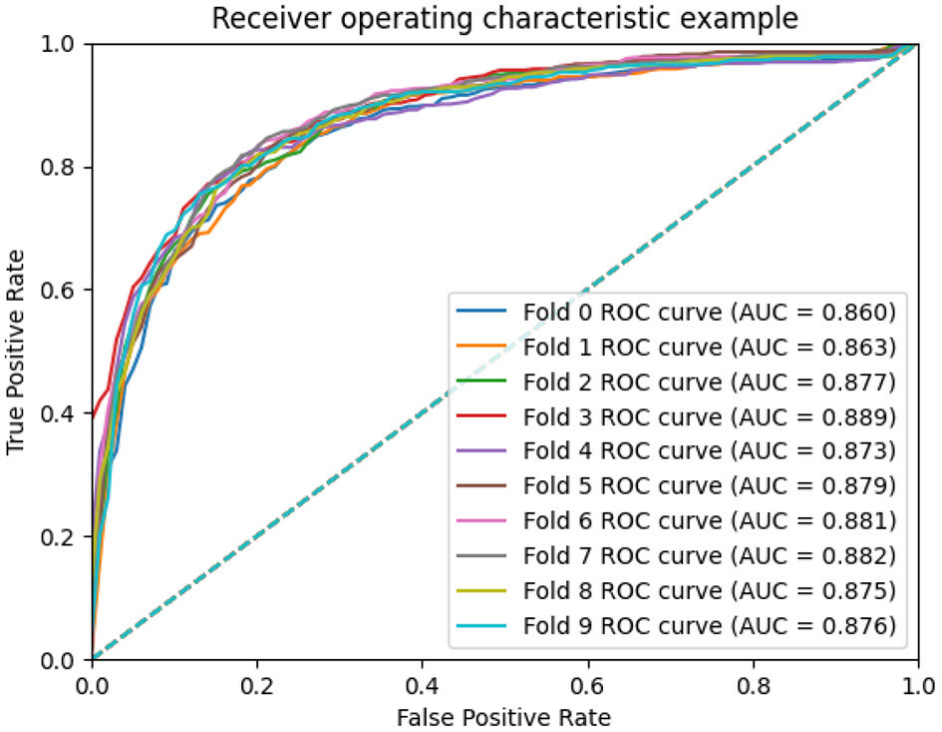
AUC performance of GCNA-MDA model on 10-fold cross-validation.

To measure the scalability of the GCNA-MDA model, we perform 5- and 10-fold cross-validation on the HMDD dataset. The results of 5-fold cross-validation are shown in Figure 3. The GCNA-MDA model achieved AUC values of 0.867, 0.878, 0.875, 0.878, and 0.867 in five experiments. The average of 5 AUCs is 0.8730, and the standard deviation is 0.00526. This shows that our model has good scalability and its performance is not easily affected by random factors. In order to further eliminate the interference of other factors, our GCNA-MDA model was subjected to a 10-fold cross-validation experiment on the HMDD dataset. Figure 4 shows the AUC performance of 10-fold cross-validation. The GCNA-MDA model achieved AUC values of 0.860, 0.863, 0.877, 0.889, 0.873, 0.879, 0.881, 0.882, 0.875, and 0.876 in 10 experiments. It can be calculated that the average value of the AUC indicator is 0.8755, and the standard deviation is 0.00561. We can find that there is only a difference of 0.0003 between the means of the two groups of experiments, and a difference of 0.00338 between the standard deviations of the two groups. Such variance is perfectly acceptable because random sampling is not controllable. It shows that the performance of the GCNA-MDA model is very stable, and it also shows that its accuracy will not be affected by random sampling. In addition, this may also be due to the local sampling strategy adopted in our research, so that the distribution and ratio of positive and negative samples tend to be similar at the same time.

### 3.4. Evaluation of different forecasting methods

Table 2 compares the performance of two autoencoder-based methods. The DFELMDA model (Liu et al., 2022b) employs autoencoders for feature extraction and random forests for miRNA-disease association prediction. While it performs well on the AUC indicator, its performance on other indicators is unsatisfactory, possibly due to overfitting caused by random forests. Moreover, the extreme imbalance of positive and negative samples further contributes to the low indicators. In contrast, the GCNA-MDA model performs consistently across all indicators, likely because it utilizes GCN in the prediction module, which effectively incorporates topological information. Additionally, we address the issue of imbalanced samples by maintaining a 1:1 ratio of positive and negative samples.

**Table 2:** Performance comparison of two models using autoencoders (%).

**Models**	**AUC**	**AUPR**	**MCC**	**F1- score**	**Precision**
	86.66	86.80	55.90	73.97	85.78
	87.80	88.42	58.33	73.61	90.24
GCNA-MDA	87.54	88.60	58.77	74.57	89.33
	87.75	87.99	57.19	75.49	85.19
	86.73	87.23	53.51	69.86	88.43
Average	87.30	87.81	56.74	73.50	87.80
DFELMDA (Liu et al., 2022b)	95.56	58.49	13.17	14.23	20.57

### 3.5. Case analysis

In order to verify the validity of our model, we conduct case analysis of 10 related diseases on the miRNA numbered hsa-mir-29a. In a more detailed operation, we selected the best model parameters in a 5-fold cross-validation experiment, and then selected these diseases in Table 3 as an external test set to predict the association with hsa-mir-29a. We picked 7 positive samples associated with hsa-mir-29a and 3 negative samples not associated with hsa-mir-29a. Table 3 presents the results of the case analysis. By comparing the results in the original database, the GCNA-MDA model correctly predicted all associations in the case analysis. This shows that the GCNA-MDA model does have certain reliability and can be further used as a reference for disease prediction.

**Table 3:** A case study of the association of miRNA named hsa-mir-29a with various diseases.

**Diseases**	**Predicted**	**Diseases**	**Predicted**
Carcinoma, hepatocellular	Verified	Heart failure	Verified
Liver neoplasms	verified	Cerebral infarction	Unverified
Influenza, human	verified	Colonic neoplasms	Verified
Scleroderma, localized	Verified	Gerstmann-Straussler-Scheinker disease	Verified
Skin neoplasms	Unverified	Carcinoma, Small cell	Unverified

We also performed a case analysis of the model on the disease side. For instance, we analyzed miRNAs potentially associated with Renal Cell-related cancer. Table 4 presents the analysis results, indicating that the GCNA-MDA model accurately identifies miRNAs associated with the disease by comparing databases. Thus, our model is effective for case studies involving both miRNAs and diseases.

**Table 4:** A case study of the association of disease named Carcinoma, Renal Cell with various miRNAs.

**miRNAs**	**Predicted**	**miRNAs**	**Predicted**
hsa-mir-132	Verified	hsa-mir-1303	Verified
hsa-mir-378b	Verified	hsa-mir-378e	Verified
hsa-mir-141	Verified	hsa-mir-218	Verified
hsa-mir-19b	Verified	hsa-mir-196b	Unverified
hsa-mir-498	Unverified	hsa-mir-3196	Verified

## 4. Conclusion

In this paper, a GCNA-MDA model that accurately predicts miRNA-disease associations is proposed based on dual autoencoders and GCN. We proposed a novel feature integration strategy based on the combination of multi-way data such as association similarity and feature similarity. This allows for a more complete initial representation of the node. Furthermore, we further perform feature extraction on these initial node representations with higher dimensions based on the dual-autoencoder. The self-supervised learning strategy alleviates the problem of insufficient positively correlated data, resulting in a more robust initial node embedding matrix. Finally, based on GCN, we perform corresponding aggregation operations on all miRNAs and disease nodes, and perform association prediction tasks. We constructed comparative experiments and scalability experiments to verify the effectiveness and scalability of our model. The case analysis of hsa-mir-29a shows that the GCNA-MDA model has certain practical significance.

## Data availability statement

The original contributions presented in the study are included in the article/supplementary material, further inquiries can be directed to the corresponding authors. Our data and code are available at https://github.com/Lqingquan/GCNA-MDA.

## Author contributions

All authors listed have made a substantial, direct, and intellectual contribution to the work and approved it for publication.
